# Whole-brain characterization of apoptosis after sevoflurane anesthesia reveals neuronal cell death patterns in the mouse neonatal neocortex

**DOI:** 10.1038/s41598-023-41750-w

**Published:** 2023-09-07

**Authors:** Julie Areias, Chrystelle Sola, Yan Chastagnier, Julien Pico, Nathalie Bouquier, Christophe Dadure, Julie Perroy, Vivien Szabo

**Affiliations:** 1 IGF, University of Montpellier, CNRS, INSERM, Montpellier, France; 2 Montpellier University Hospital, 191 Av. du Doyen Gaston Giraud, 34295 Montpellier Cedex 05, France

**Keywords:** Cell death in the nervous system, Brain

## Abstract

In the last two decades, safety concerns about general anesthesia (GA) arose from studies documenting brain cell death in various pharmacological conditions and animal models. Nowadays, a thorough characterization of sevoflurane-induced apoptosis in the entire neonatal mouse brain would help identify and further focus on underlying mechanisms. We performed whole-brain mapping of sevoflurane-induced apoptosis in post-natal day (P) 7 mice using tissue clearing and immunohistochemistry. We found an anatomically heterogenous increase in cleaved-caspase-3 staining. The use of a novel P7 brain atlas showed that the neocortex was the most affected area, followed by the striatum and the metencephalon. Histological characterization in cortical slices determined that post-mitotic neurons were the most affected cell type and followed inter- and intracortical gradients with maximal apoptosis in the superficial layers of the posterodorsal cortex. The unbiased anatomical mapping used here allowed us to confirm sevoflurane-induced apoptosis in the perinatal period, neocortical involvement, and indicated striatal and metencephalic damage while suggesting moderate hippocampal one. The identification of neocortical gradients is consistent with a maturity-dependent mechanism. Further research could then focus on the interference of sevoflurane with neuronal migration and survival during development.

## Introduction

Millions of infants worldwide require general anesthesia every year. In the United States of America, for instance, 15% of children undergo at least one general anesthesia before the age of three^1^. However, experimental results from the past two decades have raised serious safety concerns about the exposure of the developing brain to general anesthetic agents. One of the pioneering pre-clinical studies performed in rats showed that N-methyl-D-aspartate (NMDA) glutamate receptors blockade using ketamine during the perinatal period resulted in widespread brain neuronal cells apoptosis^2^. Since then, numerous studies have explored the impact of neonatal exposure to various anesthetic agents on brain maturation, and the potential long-term consequences on behavioral and cognitive development in various animal species as well as in humans^3–5^. Alerted by these data, the Food and Drug Administration (FDA) posted a drug safety communication^6,7^ explicitly warning against high-risk exposure to anesthesia during infancy, defined as either repeated or lasting more than 3 h. Approximately 25% of children under 3 years of age who undergo a procedure fall into this category^1^.

Most pharmacological agents commonly administered to induce general anesthesia, including volatile anesthetic agents widely used in the pediatric population, interfere with y-aminobutyric acid (GABAergic), glutamatergic, or both type of synaptic transmission, either by increasing GABA-A receptor open-state probability, or blocking NMDA receptors^8–10^. Regardless of the underlying cellular mechanisms, neurodegeneration after exposure to GABA-A receptors agonists and NMDA receptors antagonists is now supported by a large corpus of data, and presents some interesting features (for reviews, see^3,10–12^). First, it appears to be age-dependent. The period of greatest vulnerability to anesthetic agents’ exposure corresponds to the brain growth spurt, i.e. the peak of rapid synaptogenesis, occurring at approximately 7 days of life in rodents^2,13–19^. Physiological brain maturation involves a process of programmed cell death, i.e. developmental apoptosis^20^, in which the GABAergic and glutamatergic systems play a crucial role in modulating neuronal progenitor proliferation, migration, and maturation^21–23^. Therefore, anesthetic agents' interference with GABAergic and/or glutamatergic transmission during early brain development is likely to result in some perturbation of programmed cell death^4,10,23^. Second, toxicity also appears to be dose-dependent^3,13,24–28^ and the combination of a GABA-A receptor agonist and a NMDA receptor antagonist has been reported to be another worsening factor^29^. Finally, this increased apoptosis has been associated with functional abnormalities, including long-term cognitive and behavioral impairment in rodent models^11,17,24,27–30^, and similar findings have been replicated in non-human primates^5,31^, supporting the pathological nature of this cellular loss and legitimating the concern for young children.

Interestingly, the anatomical distribution of anesthesia-induced apoptosis does not appear homogeneous throughout the brain^3,12,15–18,27,32^. However, interpretation of the anatomical and histological variability of the reported damage is impaired, for one part by the use of different animal models including rodents, guinea pigs, piglets and non-human primates^19,33–37^, and for the other by the use of different anesthetic agents.

Therefore, even though the pro-apoptotic effects of anesthetic agents seem well-established and despite years of extensive research, the precise mechanism of anesthesia-induced neurotoxicity and the subsequent potential implications for the practice of pediatric anesthesia are still debated. Research efforts remain necessary in order to better understand their underlying mechanisms and then optimize anesthesia drugs and methods used to limit their potential side-effects. We propose that the effects of sevoflurane, the most widely inhaled anesthetic agent used in pediatric population, should be thoroughly examined. In this study, we present an unbiased anatomical mapping of cell apoptosis induced by sevoflurane exposure during postnatal brain development in mice, followed by histological characterization of cells in the areas identified as most affected.

## Results

### Whole brain mapping and quantification of anesthesia-induced apoptosis

**Figure 1:**
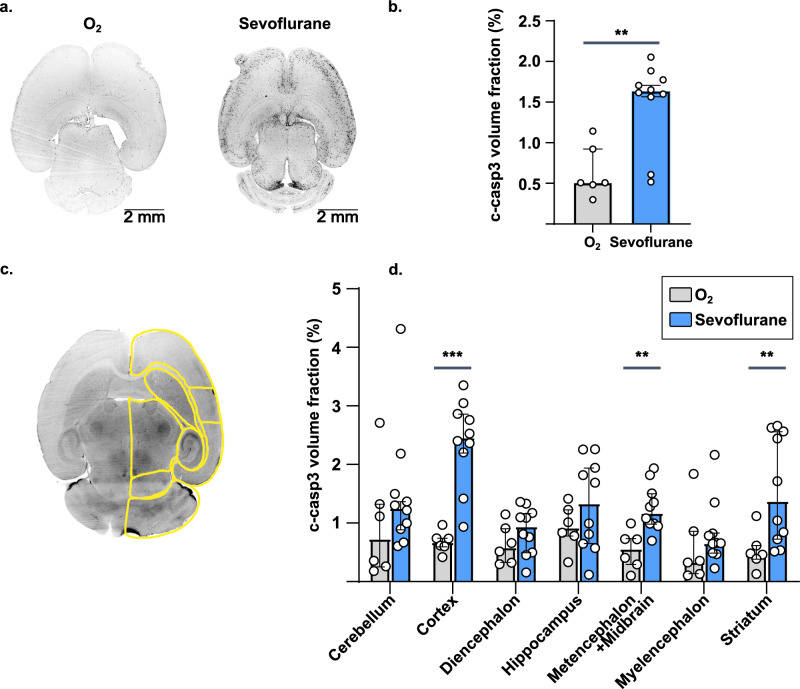
Whole brain mapping of apoptosis after sevoflurane anesthesia. (**a**) Examples of fluorescence images of c-caspase-3 immunostaining of mice brains after oxygen or sevoflurane exposure at P7. (**b**) Whole-brain quantification of c-caspase-3 staining volume fraction. (**c**) Example view of the 3D atlas used for anatomical segmentation of P7 brains. (**d**) Anatomical quantification of c-caspase-3 staining volume fraction at P7. **p ≤ 0.01, ***p ≤ 0.001. Data are presented as median (± IQR). n = 6 (O_2_), 10 (Sevoflurane)*.*

We first aimed to provide whole-brain maps of sevoflurane-induced apoptosis in P7 mice. In the control group, c-caspase-3 immunostaining was detected in 0.507 [0.482–0.922] % of the brain volume. After exposure to sevoflurane, the volume fraction of c-caspase-3 increased significantly, reaching 1.63 [1.56–1.77] % (*p* = 3.00 × 10^–3^) of brain volume (Fig. 1a,b). These results suggest a high sensitivity of the developing brain to anesthesia exposure resulting in an increase in apoptosis.

### Anatomical distribution of neonatal anesthesia-induced apoptosis

In order to characterize the anatomical distribution of c-caspase-3 positive cells after sevoflurane exposure during the neonatal period, we developed a numerical atlas of the P7 mouse brain for semi-automated registration and segmentation (Fig. 1c and Methods). The following seven brain regions have been segmented: myelencephalon, metencephalon/midbrain, diencephalon, hippocampus, neocortex, striatum, and cerebellum. After exposure to sevoflurane, the c-caspase-3 volume fraction was significantly increased in the neocortex (2.47 [2.21–2.86] versus 0.676 [0.602–0.740] %; *p* = 4.99 × 10^–4^), as well as in the striatum (1.38 [0.723–2.57] vs. 0.471 [0.390–0.616] %; *p* = 1.10 × 10^–2^), and in the metencephalon (1.18 [0.991–1.51] vs. 0.564 [0.293–0.727] %; *p* = 7.49 × 10^–3^) (Fig. 1d). In other brain regions, the c-caspase-3 volume fraction showed a tendency to increase, with no statistical significance. These results suggest that, despite intense and diffuse apoptosis after neonatal sevoflurane exposure, vulnerability is brain region dependent. In the present results, the neocortex was particularly affected.

### Histological characterization of cortical apoptotic cells

Results from whole-brain mapping showed that the brain area most severely affected by sevoflurane-induced cell apoptosis was the neocortex. We then focused on characterizing the spatial cortical distribution and the cytological nature of the apoptotic cells affected by anesthesia-induced apoptosis.

**Figure 2:**
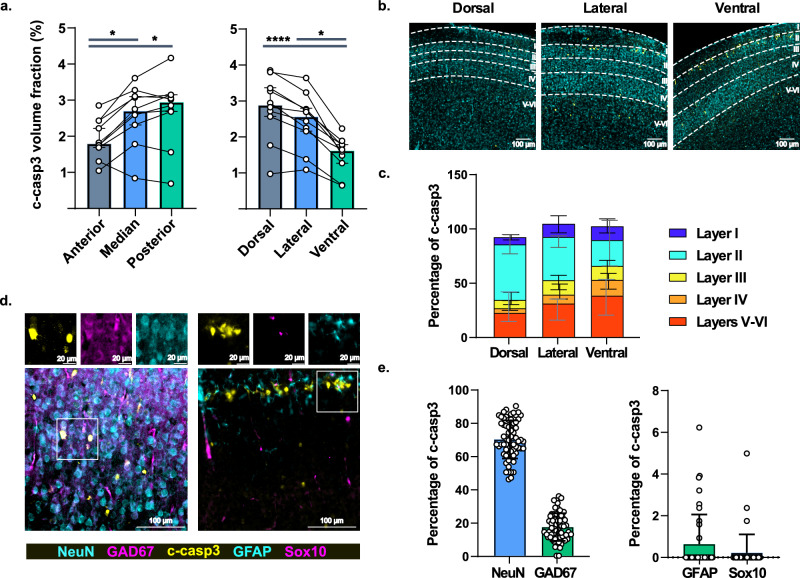
Characterization of neocortical apoptosis after sevoflurane anesthesia at P7. (**a**) Anatomical gradients of c-caspase-3 staining volume fraction, n = 10. (**b**) Examples of fluorescence images acquired in the neocortex showing the intracortical distribution of c-caspase-3 positive cells. (**c**) Quantification of the distribution of intracortical c-caspase-3 positive cells, n = 14 (dorsal), 15 (lateral), 10 (ventral). (**d**) Examples of fluorescence images acquired after immunostainings for intracortical characterization of c-caspase-3 positive cells. Boxes show higher magnification images in single channels. (**e**) Quantification of the fraction of cortical cells co-staining with c-caspase-3, n = 65 (left), 41 (right). *p ≤ 0.05; ****p ≤ 0.0001. Data are presented as median (± IQR) except in e. for which data are presented as mean (± s.d.).

First, immunostainings showed macroscopic cortical antero-posterior and ventro-dorsal gradients of apoptotic cells. The median volume fraction of c-caspase-3 labeled cells was 1.79 [1.69–2.43] % in the anterior cortex, significantly lower than in the medial (2.70 [2.32–3.11]; *p* = 4.17 × 10^–2^) and posterior cortices (2.94 [2.68–3.14] %; *p* = 1.10 × 10^–2^). Similarly, the volume of c-caspase-3 labeled cells was lower in ventral cortex (1.62 [1.26–1.78] %) than in the lateral (2.56 [2.15–2.77] %; *p* = 4.17 × 10^–2^) and the dorsal (2.88 [2.56–3.35] %; *p* = 6.45 × 10^–5^) ones (Fig. 2a). Second, this experiment also highlighted intracortical interlaminar gradients. In the dorsal cortex, the majority of c-caspase-3 positive cells, accounting for 51.1 [43.5–60.0] %, were located in superficial layer II (Fig. 2b,c). In contrast, in the ventral cortex, deep layers V-VI contained the greatest proportion of c-caspase-3 positive cells, 38.1 [21.4–50.0] %, whereas superficial layer II was less affected, representing only about one quarter of the c-caspase-3 labeled cells. No difference was found when considering the antero-posterior axis. These results support differential vulnerability to sevoflurane according to cortical areas, the superficial layer of the postero-dorsal part of the neonatal neocortex being the most affected in the current work. These could be related to differential timing in neurogenesis and maturation in a brain region-dependent manner.

Finally, we characterized the cell types of c-caspase-3 positive cells using co-immunostainings (Fig. 2d). The vast majority of apoptotic cells, accounting for 69.7%, were NeuN positive, 24.6% of which were co-labeled with the GABAergic marker GAD67 (Fig. 2e). Glial cell staining represented only 0.87% of c-caspase-3 positive cells, divided into 0.645% astrocytic GFAP and 0.223% oligodendrocytic Sox10 positive cells. The remaining 30% of c-caspase-3 positive cells did not stain with any of the above-mentioned markers. These data suggest that sevoflurane-induced apoptosis mainly affected post-mitotic neurons, of which 24.6% were GABAergic neurons. In contrast, glial cells, both oligodendrocytes and astrocytes appear to be almost not affected.

## Discussion

In order to establish a complete and accurate mapping of the potential cellular damage induced by anesthetics, we used an innovative whole-brain approach. In contrast to the vast majority of previous studies that have explored anesthetic-induced neurotoxicity, the clarification method allowed us to obtain images of entire intact mice brains and thus to avoid anatomical bias induced by incomplete tissue sampling after slicing. Our unbiased whole-brain immunohistochemical approach revealed a high sensitivity of the immature brain (P7) to sevoflurane-induced apoptosis, particularly affecting the neocortex. Moreover, the distribution of c-caspase-3 cells was not uniform in the neocortex following a cortical gradient both antero-posteriorly and ventro-dorsally, with the postero-dorsal part of the neonatal neocortex mostly affected in the current work. Furthermore, a radial intracortical gradient was observed such that the deep layers (V-VI) seemed to be more affected in the ventral cortex, while the superficial layers (II) were mainly impacted in the dorsal one. Finally, cytological characterization showed that most of the apoptotic cells were post-mitotic neurons, of which about one quarter were GABAergic cells.

The method used in the present studies presents two key advantages. First, it is free of anatomical bias, since entire brains are screened for apoptotic cells. This top-down approach allows secondary focus on regions of interest, defined a posteriori, in contrast to a priori targets in tissue slices, usually limited in number. Second, the analysis pipeline is largely automated and user-independent. This ensures high reproducibility, and also frees a significant amount of time for the experimenter. However, it should be noted that sensitivity usually achieved in slices is probably higher than the one reached here. One could increase this sensitivity, first, by using a higher acquisition resolution, but hence getting a larger data volume in a longer acquisition time, and second, by setting a threshold for signal at a lower value, while accepting a smaller specificity.

The anatomical distribution of apoptotic cells depicted in this study is consistent with the reported data collected from brain slices, indicating particular vulnerability of the neocortex. However, it has been shown that a variety of regions can be more or less affected by anesthesia-induced cell apoptosis^3,12,15–18,27,32^, including cortical and subcortical areas to various extents. In the present report, the anatomical distribution of c-caspase-3 staining was significantly increased in the striatum after neonatal sevoflurane exposure, in contrast to the results of a similar study that exposed mice to a general anesthetic in the early postnatal period^17^. Additionally, the hippocampal apoptotic damage reported here appeared to be limited compared to data published elsewhere^15^. These discrepancies may be due to a different anesthesia protocol including dosing, exposure time and pharmacological agents. Indeed, in the two studies mentioned above, isoflurane, the other commonly used volatile anesthetic in clinical practices, was used in contrast to sevoflurane in the present study. In fact, differential neuronal cell death after exposure to sevoflurane compared to isoflurane has been previously demonstrated, suggesting a plausible explanation for these contrasting results^38^. In addition, slight variations in the age of exposure between studies or use of different mice strains could lead to this sort of interstudy variability. Finally, these disparities could also arise from incomplete sampling of sliced brain. The unbiased histological approach used here allows for whole brain screening, without preconceived expectations. This facilitates further analysis of regions of particular interest. In the present work, we focused on the area most affected by sevoflurane-induced apoptosis.

As reported in various data collected from brain slices, the present whole-brain approach indicates a particular vulnerability of the neocortex to sevoflurane-induced apoptosis^2,15,17,27,32^. We observed cortical gradients of c-caspase-3 both along the antero-posterior and ventro-dorsal axes, and also between different cortical layers. These patterns could be related to differential brain maturation in a region-dependent manner and related to physiological developmental apoptosis. It is commonly accepted that during the two first postnatal weeks in rodent, apoptosis is a physiological phenomenon crucial for proper brain development^20,39,40^. This programmed cell death is known to affect cortical neurons in an area- and layer-dependent manner such that developmental apoptosis does not appear homogeneous between functionally diverse neocortical areas or even between layers within the same cortical area^40–42^. Previous studies investigated whether anesthesia-induced apoptosis affects the same cell population as the one undergoing physiological developmental apoptosis, focusing on primary somatosensory cortex. The authors reported that cell death first affected the deep layer V at P5 and shifted to the superficial layers II–IV by P9^43,44^. Furthermore, while neuronal apoptosis was significantly increased in mice exposed to intravenous or volatile anesthesia, the histological patterns appeared similar to the physiological developmental ones. It would therefore seem that exposure to anesthetic agents pathologically increases physiological cell death^45,46^. Our data also suggest so, reporting similar interlayer cortical apoptosis gradients after sevoflurane exposure in the neonatal period.

We furthermore found that these patterns were not homogeneous across the whole neocortex and depended on the cortical area. Such regional disparities may result from spatial differences in the maturation processes and/or in the activity in different brain areas ^42^. It is plausible that anesthetic agents would affect the excitatory/inhibitory balance and trigger neuronal death^4,10,12,23,40^. For instance, GABA_A_-R are thought to generate excitatory post-synaptic currents during the early development, and only later shift to inhibitory ones depending on chloride ion Nernst potential^3^. Furthermore, NMDA-R blockade could lead to rapid up-regulation of their membrane expression^4,10^. Both mechanisms could involve excitability dysregulation in anesthesia-induced cell death. Besides, it has been convincingly shown that GABA_A_-R activation can impact neuronal radial migration^22,47^. The above-mentioned phenomena would increase neuronal death in a maturation-dependent manner and lead to the differential patterns observed in this study.

Finally, we found that post-mitotic neurons accounted for the vast majority of apoptotic cells. These results are consistent with the literature^2,10,32,48^, since most studies indicate that neuronal cells are particularly affected by anesthesia-induced apoptosis, with a variable proportion of GABAergic neurons involved^3,32^. In concordance, our data showed that 24.6% of apoptotic post-mitotic neurons were GABAergic. Although this rate is almost 10 times higher than reported elsewhere^48^, it is consistent with other results^44^. These differences may arise from the important dependance on age of GABAergic neurons susceptibility to sevoflurane^44^. Significant damage to glial cells, particularly oligodendrocytes in the white matter has also been reported in fetal and neonatal non-human primate brains after exposure to volatile anesthesia while astrocytes appeared to be spared^3,33–35,48^. These contrasting results can stem from various causes. First, we did not perform quantification in the white matter since the initial whole-brain screening did not point towards it. Second, periods of cell proliferation and differentiation appear to increase sensitivity to anesthesia-induced neurotoxicity, as evidenced by the diffuse neuronal apoptosis associated with exposures during the first week of life in rodents. In contrast to neurons, glial cells proliferation occurs later, mainly between the second and third week of life in rodents^49^. Finally, the few publications on anesthesia-induced oligodendrocyte toxicity have been performed in non-human primates whose timing of brain development and maturation processes is different from that of rodents. These elements could explain why oligodendrocytes were virtually unaffected in the present study. Finally, we were not able to determine the cell type of about 30% of the apoptotic cells. We can only suspect that these were undergoing differentiation and did not express the usual markers.

The loss of neurons by apoptosis described here after exposure of the developing brain to sevoflurane has been described for all commonly used anesthetic drugs, including propofol, other volatile anesthetics (isoflurane, desflurane), ketamine, benzodiazepines. Furthermore, this increased apoptosis has been associated with functional abnormalities, including long-term cognitive and behavioral impairment in rodent models^11,17,24,27–30^, as well as in non-human primates with similar findings replicated^5,31^. Although neuroapoptosis is a normal part of mammalian development, these extensive experimental data support the pathological nature of this cell loss. However, how these results can translate to human remains uncertain. Data from human trials, the vast majority of which being retrospective and presenting numerous confounding factors related to patient comorbidities, duration and surgery type, and environmental factors, are mixed, some of them showing neurodevelopmental disorders in children exposed to general anesthesia in their infancy, while others found no association^50^.

Further research is still needed to better understand the mechanisms of neuronal loss and dysfunction observed after exposure to anesthesia and their possible long-term consequences. The present work shows unbiased quantification of sevoflurane anesthesia-induced apoptosis as well as histological identification and characterization in the neocortex, the most affected brain region. This whole-brain screening provides a valuable step before more detailed explorations, since anatomical disparities in anesthesia-induced cellular modifications are now established, down to region-dependent impact on the transcriptome and the epigenome^51,52^. Next steps would include identification of signaling pathways, from membrane receptors to the activation of caspase cascades encompassing critical kinases such as ERK, shown to be downregulated by sevoflurane in non-GABAergic cells^53^. Finally, testing of potential neuroprotective compounds, free of GABAergic or glutamatergic effects such as alpha-2 adrenoceptor agonists (i.e. dexmedetomidine)^10^ will be of promising interest to increase our understanding of the mechanics of general anesthesia with potential direct impact on clinical practice.

## Methods

All methods are reported in accordance with ARRIVE guidelines.

### Animals

All animal procedures were conducted in accordance with the European Communities Council Directive, supervised by the IGF institute’s local Animal Welfare Unit (national registration number A34-172-41, procedure number HCR#140). Animals exposed to experiment were obtained from experimentally naïve timed-pregnant C57Bl/6 J mice (Janvier Labs). The offspring remained housed with the dam until experiment (P0 defined as the day of birth) and maintained in a 12-h light/dark cycle (lights on 7:30 AM to 7:30 PM), in stable conditions of temperature (22 ± 2 °C) and humidity (60%), with food and water provided ad libitum. The weight and general appearance of all mice were carefully monitored throughout the experiment. Efforts were made to minimize the number of animals used and their suffering.

### Anesthesia protocol

Litter-matched mice pups were randomly assigned to the sevoflurane or control (no anesthesia) groups. In the sevoflurane group, animals were exposed at P7 to 3% sevoflurane (agent specific vaporizer in O_2_ 100% as gas carrier) for 3 consecutive periods of 2 h separated by the amount of time necessary for the mice to regain righting reflex (approximately 5 min) in a pre-warmed Plexiglas chamber kept on a heating plate at 37 °C. In the non-exposed group, the animals were placed under identical condition in O_2_ 100% for the same duration and at the same developmental stage (i.e. P7). The gas flow was 1 L.min^−1^. We did not monitor the animals’ body temperature nor arterial blood gas. However, we chose an anesthetic protocol consistent with the literature on neonatal reporting no alteration of pH, arterial oxygen and carbon dioxide tensions, arterial oxygen saturation or cerebral blood flow in anesthetic conditions similar to that used in the present study^54,55^. In addition, it has been convincingly shown that body temperature does not correlate with c-caspase-3 staining^14^.

Immediately following treatment, mice were euthanized by decapitation, brains were carefully removed and postfixed overnight at 4 °C in 4% paraformaldehyde (PFA) in a sodium phosphate buffer (PBS).

### Whole-brain clearing and immunolabeling of c-caspase-3 positive cells

The iDISCO + (immunolabeling-enabled three-dimensional imaging of solvent-cleared organs) method was used for whole brain immunolabeling according to the original published protocol^56^, briefly described here:

*Tissue preparation*: The fixed samples were incubated in methanol/H_2_O series in gradually increasing concentrations of methanol until complete dehydration and incubated overnight in 67% dichloromethane/33% methanol. Samples were then bleached with fresh 5% H_2_O_2_ in methanol at 4 °C overnight. Subsequently, the samples were rehydrated in methanol/H_2_O series in gradually decreasing concentrations of methanol. Before immunolabeling, samples were incubated in permeabilization solution (0.2% Triton X-100/20% DMSO/0.3 M glycine) at 37 °C overnight, then blocked in PBS/0.2% Triton X-100/10% DMSO/6% Donkey Serum for 2 days at 37 °C.

*Immunolabeling*: Pre-treated samples were incubated in rabbit anti-cleaved caspase-3 primary antibody (1:500, Cell Signaling 9661) in PBS 0.2% Tween-20 with heparin (PTwH)/5% DMSO/3% Donkey Serum solution at 37 °C for 10 days. Samples were then washed in PTwH for 1 day, then incubated with a donkey anti rabbit Alexa 647 secondary antibody (1:500–Thermo Fisher A-315) in PTwH/3% Donkey Serum solution at 37 °C for 10 additional days. Samples were finally washed in PTwH for 2 days before clearing.

*Clearing*: The brains were dehydrated as previously described in a methanol/H_2_O series at room temperature and incubated in 67% dichloromethane/33% methanol for 3 h with shaking. The samples were then washed in 100% dichloromethane twice and finally transferred to dibenzyl ether until imaging. N.B. Organic solvents should be handled under a chemical hood. Dibenzyl ether is a skin irritant exhibiting moderate toxicity, it should be handled with gloves.

### Immunohistochemistry on brain slices

*Tissue preparation*: The fixed samples were rinsed with PBS and free-floating sections of 30 µm thickness were obtained using a Leica vibratome VT1000S.

*Antibodies*: To determine the phenotype of cells identified as apoptotic, different co-labelings were applied using the following primary antibodies: Rabbit polyclonal anti-cleaved caspase-3 (1:400–Cell Signaling); mouse monoclonal anti-glutamate- decarboxylase isoform 67 (GAD67, 1:1000–Millipore), anti-SRY-box 10 (Sox10, 1:1000–Novus); chicken polyclonal anti-neuronal nuclei (NeuN–1:500–Synaptic Systems), anti-GFAP (1:1000 Abcam). Following fluorophore-labeled secondary donkey-antibodies were used: anti-rabbit Cy3-conjugated (1:500–Jackson ImmunoResearch), anti-chicken Alexa 680-conjugated (1:500–Jackson ImmunoResearch), anti-mouse Alexa 488-conjugated (1:500–Invitrogen).

*Immunohistochemistry*: Floating sections were blocked for 1 h at room temperature with PBS, 5% (v/v) Normal Donkey Serum, 0.5% (w/v) Bovine Albumin Serum (BSA), 0.1% (v/v) Tween-20 (T20) before incubation overnight at 4 °C with primary antibodies diluted in antibodies solution (PBS 1X, 0.5% BSA, 0.1% T20). After washing three times in PBS, the sections were incubated for 1 h at room temperature with complementary secondary antibodies diluted in antibodies solution. Nuclei were stained with Hoechst-33342 (Sigma) solution diluted at 1:1000 in PBS for 10 min. After three PBS washes, sections were mounted in Dako mounting medium (Dako).

### Whole brain 3D light sheet fluorescence microscope imaging

Whole-brain samples were imaged in an axial orientation on the Ultramicroscope (Miltenyi Biotec) setup equipped with a sCMOS camera and a 2 × /0.5 objective lens (MV PL-APO – Olympus) with a 5.7 mm working distance. Whole brains were imaged in a chamber filled with dibenzyl ether. The ImSpector Pro control software was used. Images were acquired at 0.63 × magnification with an exposure time of 100 ms in a z-stack with 3.5 μm intervals (0.035 NA–7 µm thickness light-sheet). Data were acquired in two channels: Autofluorescence and antibody-specific channel. Autofluorescence was acquired at excitation wavelength of 488 nm and emission wavelength of 525/50 nm. Antibody-specific channel was acquired at excitation wavelength of 639 nm and emission of 680/30 nm.

### Fixed slices microscopic imaging

Immunofluorescence two dimensional images were generated using a Leica SP8-UV laser scanning confocal microscope (PL-APO 10X, 0.4 NA, imaging medium Leica) for sections co-stained with NeuN and GAD67, or a Zeiss AxioImager Z1 microscope (PL-NEO 10X, 0.3 NA) for sections co-stained with Sox10 and GFAP. Emission and excitation filters proper to each fluorophore were used sequentially: Cy3 excitation 545/25 nm and emission 605/70 nm, Alexa 680 excitation 665/45 nm and emission 725/50 nm, Alexa 488 excitation 475/40 nm and emission 530/50 nm.

### Image analysis

*Three-dimensional image processing*. Fiji software was used to register image stacks onto each other. To do so, a line was drawn for each brain between two easily recognizable landmarks. The direction of the line was used to rotate the images, so all brains have the same orientation. The length of the line was used to stretch the images, so all brains have the same size. Finally, the position of the line gave a xyz positional reference. All brains were assumed to be flat landed and have no tilt around the x and y axis of the images.

*Atlas development*: Fiji software was used to create an atlas of the P7 brain mouse. One brain was chosen, and regions were manually drawn on 50 horizontal slices for most regions except for dorsal, lateral and ventral cortex which were drawn on 30 coronal slices. Matlab software was then used to reconstruct a volumetric atlas using interpolation and process the images.

*Three-dimensional image analysis*: first, images were segmented with a user defined threshold to separate the brain from the background on a gaussian blurred version of the image (σ_xy_ = 10 vx). Noise sigma was determined using 16th and 84th percentile of the distribution of voxel values inside the brain (+/− 1 σ). Images were then high pass (subtraction of a gaussian blurred version of the image, σ_xy_ = 6 vx), and low pass (gaussian blur, σ_xy_ = 1 vx) filtered. After this process, every voxel with a value above 2 noise sigma was considered a c-caspase-3 positive voxel. Division of positive voxels by total voxels in each region gave the final results, presented in Supplementary file 1.

*Two-dimensional image analysis*. Fiji software was used for image processing and quantification of immunochemistry staining. Cortical layers were determined on Hoechst-33342 labeled images. General cell counter plugin (https://github.com/ychastagnier/General-Cell-Counter) was used for c-caspase-3 expressing cells quantification (Image pixel size (µm): 0.645, plugin parameters: Threshold method: Auto Local Threshold–Mean, Gaussian high pass sigma (µm): 8, Median filter radius (px): 0, Local Threshold radius (px): 25, Offset: -30, Cell area range (µm^2^): 15–140, Cell circularity range: 0.1–1.0, Minimal distance (µm): 1). Cells co-expressing the c-caspase-3 with other markers were manually counted. Results are presented in Supplementary file 1.

### Statistical analysis

Statistical analyses were performed using Graph Pad Prism software, version 9.5.1 (Graph Pad Software, La Jolla, CA, United States). The normality of distribution of continuous variables was tested by the Shapiro–Wilk test. Two-tailed Mann–Whitney U test was used to compare means of 2 groups of independent variables. Two-tailed Friedman’s one-way analysis of variance (ANOVA) followed by Dunn test were used to compare the three paired conditions. Alpha = 0.05 for all statistical tests.

Data are presented as median [IQR] except for histological characterization, data are presented as mean [s.d.]. A value of *p* < 0.05 was considered significant.

### Supplementary Information

Supplementary Information.

## Data Availability

Raw images are available upon request. Data resulting from image analysis are presented in the supplementary file.

## References

[1] Shi Y (2018). Epidemiology of general anesthesia prior to age 3 in a population-based birth cohort. Pediatr. Anesth..

[2] Ikonomidou C (1999). Blockade of NMDA receptors and apoptotic neurodegeneration in the developing brain. Science.

[3] Istaphanous GK, Ward CG, Loepke AW (2010). The impact of the perioperative period on neurocognitive development, with a focus on pharmacological concerns. Best Pract. Res. Clin. Anaesthesiol..

[4] Gascoigne DA, Serdyukova NA, Aksenov DP (2021). Early development of the GABaergic system and the associated risks of neonatal anesthesia. Int. J. Mol. Sci..

[5] Andropoulos DB (2018). Effect of anesthesia on the developing brain: infant and fetus. Fetal Diagn. Ther..

[6] US Food and Drug Administration. Drug Safety and Availability—FDA Drug Safety Communication: FDA Review Results in New Warnings about Using General Anesthetics and Sedation Drugs in Young Children and Pregnant Women. (2016).

[7] US Food and Drug Administration. FDA Drug Safety Communication: FDA Approves Label Changes for Use of General Anesthetic and Sedation Drugs in Young Children. (2017).

[8] Franks, N. P. & Lieb, W. R. Molecular and cellular mechanisms of general anaesthesia. *Nature* **367**, (1994). 10.1038/367607a0 7509043

[9] Campagna JA, Miller KW, Forman SA (2003). Mechanisms of actions of inhaled anesthetics. N. Engl. J. Med..

[10] Walters JL, Paule MG (2017). Review of preclinical studies on pediatric general anesthesia-induced developmental neurotoxicity. Neurotoxicol. Teratol..

[11] Loepke AW, Soriano SG (2008). An assessment of the effects of general anesthetics on developing brain structure and neurocognitive function. Anesth. Analg..

[12] Aksenov DP, Miller MJ, Dixon CJ, Drobyshevsky A (2021). Impact of anesthesia exposure in early development on learning and sensory functions. Dev. Psychobiol..

[13] Yon JH, Daniel-Johnson J, Carter LB, Jevtovic-Todorovic V (2005). Anesthesia induces neuronal cell death in the developing rat brain via the intrinsic and extrinsic apoptotic pathways. Neuroscience.

[14] Gutierrez S (2010). Is age-dependent, ketamine-induced apoptosis in the rat somatosensory cortex influenced by temperature?. Neuroscience.

[15] Deng M (2014). Brain regional vulnerability to anaesthesia-induced neuroapoptosis shifts with age at exposure and extends into adulthood for some regions. Br. J. Anaesth..

[16] Hofacer RD (2013). Cell age-specific vulnerability of neurons to anesthetic toxicity. Ann. Neurol..

[17] Maloney SE (2019). Repeated neonatal isoflurane exposures in the mouse induce apoptotic degenerative changes in the brain and relatively mild long-term behavioral deficits. Sci. Rep..

[18] Krzisch, M. *et al.* Propofol anesthesia impairs the maturation and survival of adult-born hippocampal neurons. *Anesthesiology* **118**, (2013). 10.1097/ALN.0b013e3182815948 23314165

[19] Rizzi S, Ori C, Jevtovic-Todorovic V (2010). Timing versus duration: Determinants of anesthesia-induced developmental apoptosis in the young mammalian brain. Ann. N. Y. Acad. Sci..

[20] Dikranian K (2001). Apoptosis in the in vivo mammalian forebrain. Neurobiol. Dis..

[21] Varju P, Katarova Z, Madarász E, Szabó G (2001). GABA signalling during development: New data and old questions. Cell Tissue Res..

[22] Luján R, Shigemoto R, López-Bendito G (2005). Glutamate and GABA receptor signalling in the developing brain. Neuroscience.

[23] De Lima AD, Opitz T, Voigt T (2004). Irreversible loss of a subpopulation of cortical interneurons in the absence of glutamatergic network activity. Eur. J. Neurosci..

[24] Zheng SQ, An LX, Cheng X, Wang YJ (2013). Sevoflurane causes neuronal apoptosis and adaptability changes of neonatal rats. Acta Anaesthesiol. Scand..

[25] Amrock LG, Starner ML, Murphy KL, Baxter MG (2015). Long-term effects of single or multiple neonatal sevoflurane exposures on rat hippocampal ultrastructure. Anesthesiology.

[26] Cattano D, Young C, Straiko MMW, Olney JW (2008). Subanesthetic doses of propofol induce neuroapoptosis in the infant mouse brain. Anesth. Analg..

[27] Jevtovic-Todorovic V (2003). Early exposure to common anesthetic agents causes widespread neurodegeneration in the developing rat brain and persistent learning deficits. J. Neurosci..

[28] Zhou ZB (2016). Subclinical concentrations of sevoflurane reduce oxidative stress but do not prevent hippocampal apoptosis. Mol. Med. Rep..

[29] Fredriksson A, Pontén E, Gordh T, Eriksson P (2007). Neonatal exposure to a combination of N-Methyl-D-aspartate and γ-aminobutyric acid type A receptor anesthetic agents potentiates apoptotic neurodegeneration and persistent behavioral deficits. Anesthesiology.

[30] Yu D, Jiang Y, Gao J, Liu B, Chen P (2013). Repeated exposure to propofol potentiates neuroapoptosis and long-term behavioral deficits in neonatal rats. Neurosci. Lett..

[31] Walters JL, Paule MG (2016). Review of preclinical studies on pediatric general anesthesia-induced developmental neurotoxicity. Neurotoxicol Teratol.

[32] Zhou ZWW (2011). The glutaminergic, GABAergic, dopaminergic but not cholinergic neurons are susceptible to anaesthesia-induced cell death in the rat developing brain. Neuroscience.

[33] Creeley CE (2014). Isoflurane-induced apoptosis of neurons and oligodendrocytes in the fetal rhesus macaque brain. Anesthesiology.

[34] Brambrink AM (2012). Isoflurane-induced apoptosis of oligodendrocytes in the neonatal primate brain. Ann. Neurol..

[35] Noguchi KK (2017). Isoflurane exposure for three hours triggers apoptotic cell death in neonatal macaque brain. BJA Br. J. Anaesth..

[36] Rizzi S, Carter LB, Ori C, Jevtovic-Todorovic V (2008). Clinical anesthesia causes permanent damage to the fetal guinea pig brain. Brain Pathol..

[37] Slikker W (2007). Ketamine-induced neuronal cell death in the perinatal rhesus monkey. Toxicol. Sci..

[38] Liang G (2010). Isoflurane causes greater neurodegeneration than an equivalent exposure of sevoflurane in the developing brain of neonatal mice. Anesthesiology.

[39] Oppenheim R (1991). Cell death during development of the nervous system. Annu. Rev. Neurosci..

[40] Wong FK, Marín O (2019). Developmental cell death in the cerebral cortex. Annu. Rev. Cell Dev. Biol..

[41] Rice D, Barone S (2000). Critical periods of vulnerability for the developing nervous system: evidence from humans and animal models. Environ. Health Perspect..

[42] Blanquie O (2017). Electrical activity controls area-specific expression of neuronal apoptosis in the mouse developing cerebral cortex. Elife.

[43] Wang Q (2017). Ketamine-induced apoptosis in the mouse cerebral cortex follows similar characteristic of physiological apoptosis and can be regulated by neuronal activity. Mol. Brain.

[44] Wang Q, Li Y, Tan H, Wang Y (2022). Sevoflurane-Induced Apoptosis in the Mouse Cerebral Cortex Follows Similar Characteristics of Physiological Apoptosis. Front. Mol. Neurosci..

[45] Creeley CE (2016). From drug-induced developmental neuroapoptosis to pediatric anesthetic neurotoxicity—where are we now?. Brain Sci..

[46] Nikolić M, Gardner HAR, Tucker KL (2013). Postnatal neuronal apoptosis in the cerebral cortex: Physiological and pathophysiological mechanisms. Neuroscience.

[47] Chai D, Yan J, Li C, Sun Y, Jiang H (2020). Sevoflurane inhibits neuronal migration and axon growth in the developing mouse cerebral cortex. Aging.

[48] Istaphanous GK (2013). Characterization and quantification of isoflurane-induced developmental apoptotic cell death in mouse cerebral cortex. Anesth. Analg..

[49] Zanghi CN, Jevtovic-todorovic V, Campus AM (2018). A Holistic Approach to Anesthesia-Induced Neurotoxicity and its Implications for Future Mechanistic Studies. Neurotoxicol Teratol.

[50] Salik, I. Anesthetic Neurotoxicity in Children. *Med. Res. Arch.* **10**, (2022).

[51] Ma, L. H., Yan, J., Jiao, X. H., Zhou, C. H. & Wu, Y. Q. The Role of Epigenetic Modifications in Neurotoxicity Induced by Neonatal General Anesthesia. *Front. Mol. Neurosci.* **15**, (2022). 10.3389/fnmol.2022.877263 PMC9097083 35571375

[52] Yamamoto H (2020). Transcriptome analysis of sevoflurane exposure effects at the different brain regions. PLoS ONE.

[53] Zhao X-P (2021). Early-life sevoflurane exposure impairs fear memory by suppressing extracellular signal-regulated kinase signaling in the bed nucleus of stria terminalis GABAergic neurons. Neuropharmacology.

[54] Ji M-H (2015). Pre-administration of curcumin prevents neonatal sevoflurane exposure-induced neurobehavioral abnormalities in mice. Neurotoxicology.

[55] Satomoto M (2009). Neonatal exposure to sevoflurane induces abnormal social behaviors and deficits in fear conditioning in mice. Anesthesiology.

[56] Renier N (2014). iDISCO: a simple, rapid method to immunolabel large tissue samples for volume imaging. Cell.

